# Advances in the delivery of RNA therapeutics: from concept to clinical reality

**DOI:** 10.1186/s13073-017-0450-0

**Published:** 2017-06-27

**Authors:** James C. Kaczmarek, Piotr S. Kowalski, Daniel G. Anderson

**Affiliations:** 1 Department of Chemical Engineering, Massachusetts Institute of Technology, Cambridge, Massachusetts 02139 USA; 2 David H. Koch Institute for Integrative Cancer Research, Massachusetts Institute of Technology, Cambridge, Massachusetts 02139 USA; 3 Institute for Medical Engineering and Science, Massachusetts Institute of Technology, Cambridge, Massachusetts 02139 USA; 4 Harvard and MIT Division of Health Science and Technology, Massachusetts Institute of Technology, Cambridge, Massachusetts 02139 USA

**Keywords:** Antisense oligonucleotide, Clinical trial, CRISPR, Gene editing, Gene therapy, Messenger RNA delivery, mRNA vaccine, RNA nanoparticle, Short interfering RNA delivery

## Abstract

The rapid expansion of the available genomic data continues to greatly impact biomedical science and medicine. Fulfilling the clinical potential of genetic discoveries requires the development of therapeutics that can specifically modulate the expression of disease-relevant genes. RNA-based drugs, including short interfering RNAs and antisense oligonucleotides, are particularly promising examples of this newer class of biologics. For over two decades, researchers have been trying to overcome major challenges for utilizing such RNAs in a therapeutic context, including intracellular delivery, stability, and immune response activation. This research is finally beginning to bear fruit as the first RNA drugs gain FDA approval and more advance to the final phases of clinical trials. Furthermore, the recent advent of CRISPR, an RNA-guided gene-editing technology, as well as new strides in the delivery of messenger RNA transcribed in vitro, have triggered a major expansion of the RNA-therapeutics field. In this review, we discuss the challenges for clinical translation of RNA-based therapeutics, with an emphasis on recent advances in delivery technologies, and present an overview of the applications of RNA-based drugs for modulation of gene/protein expression and genome editing that are currently being investigated both in the laboratory as well as in the clinic.

## Background

Fourteen years after the completion of the human genome project, our understanding of human genomics continues to develop at an unprecedented rate. Thanks to advances in next-generation sequencing technology, scientists have been able to identify the genetic roots of many common diseases [1]. Diseases such as cancer [2], Parkinson’s [3], rheumatoid arthritis [4], and Alzheimer’s [5] have all had many of their genetic components revealed, bringing us closer than ever to ‘personalized medicine’ [6]. Thus far, this knowledge has been well adapted for diagnostic use—but has not yet been fully translated to pharmaceutical interventions addressing the genetic defects underlying diseases. Currently, the two major structural classes of FDA-approved drugs are small molecules and proteins [7]. Small-molecule drugs, which consist predominantly of hydrophobic organic compounds, typically act by deactivating or inhibiting target proteins through competitive binding. However, the proteins that might possess such binding pockets have been estimated to account for only 2–5% of the protein-coding human genome [8]. Protein-based drugs (e.g., antibodies), by contrast, can bind with high specificity to a variety of targets or be used to replace mutated or missing proteins (e.g., delivering insulin for diabetes). However, the size and stability of proteins limit their utility towards many potential disease targets [7]. Thus, true realization of the therapeutic potential of personalized genomics requires treatments beyond those offered by current small-molecule and protein therapies.

In summary, both protein and small-molecule drugs are limited in that they cannot target every disease-relevant protein or gene. The mRNA and DNA precursors of proteins, however, are promising therapeutically in that they can be specifically targeted via Watson–Crick base pairing and, in the case of gene editing, which aims to permanently change the host’s DNA, represent an avenue to cure a genetic defect as opposed to just treating it. Over the past few decades, RNA drugs have emerged as candidates to address diseases at the gene and RNA levels. Although it has been known since 1990 that nucleic acids can be used to modulate protein production in vivo [9], therapeutic RNA delivery has been limited by a number of factors. Naked, single-stranded RNA is prone to nuclease degradation, can activate the immune system, and is too large and negatively charged to passively cross the cell membrane—and must, therefore, be provided with additional means of cellular entry and escape from endosomes, which transport extracellular nanoparticles into the cytoplasm [10]. As such, the nucleic acid delivery field has centered on the design of delivery methods and materials that will transport RNA drugs to the site of interest. In this review, we provide an overview of the current status of advances in RNA and RNA–protein therapy, with an emphasis on materials that have been developed for RNA delivery and applications of RNA-based drugs for the modulation of gene/protein expression and gene editing.

## Delivery materials and chemical modifications for RNA

### Delivery materials

**Fig. 1:**
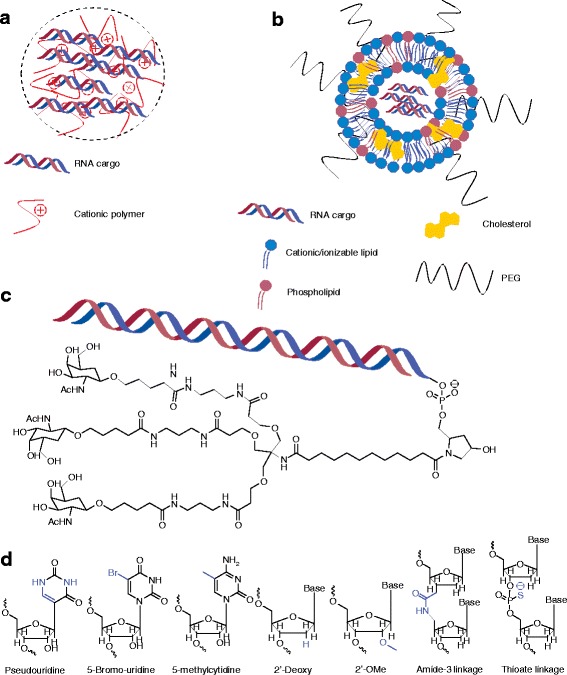
Common delivery modalities for RNA. **a** Schematic depicting polymeric nanoparticles comprising RNA and cationic polymer. **b** Schematic depicting lipid nanoparticles containing RNA, a cationic/ionizable lipid, and other hydrophobic moieties (such as cholesterol) commonly used in nanoparticle formulation. **c** Chemical structure of a tertiary conjugate between *N*-acetylgalactosamine (GalNAc) and RNA that is currently in clinical trials [38]. **d** Examples of base, sugar, and linker modifications that have been utilized to deliver nucleic acids (modified chemistry highlighted in *blue*)

Broadly speaking, RNA delivery can be mediated by viral and non-viral vectors. For viral RNA delivery, there has been a great deal of interest in engineering adeno-associated viruses to carry nucleic acid cargo [11]—however, this section will focus mainly on the development of non-viral materials (Table 1). Of the non-viral RNA delivery vehicles, nanoparticles are perhaps the most studied. Nanoparticle encapsulation of RNA physically protects nucleic acids from degradation and, depending on the specific chemistry, can aid in cellular uptake and endosomal escape. Given their high degree of chemical flexibility, polymers are commonly used materials for nanoparticle-based delivery [12]. Typically, cationic polymers are used to electrostatically condense the negatively charged RNA into nanoparticles (Fig. 1a) [13]. These positively charged groups often consist of amines that become protonated at physiological pH (p*K*a ~7.4), thought to lead to an ion imbalance that results in endosomal rupture [14, 15], although this so-called ‘proton sponge’ hypothesis has yet to be rigorously demonstrated for various materials [16]. Regardless of the exact mechanism by which polymers aid in RNA delivery, commercially available amine-containing polymers were some of the earliest non-viral materials adopted for nucleic acid delivery. Synthetic polymers such as poly-L-lysine [17], polyamidoamine [18], and polyethyleneimine [19], as well as naturally occurring polymers such as chitosan [20], have all been applied to RNA delivery, with varying levels of success. In addition, some investigators have synthesized polymers specifically for nucleic acid delivery. Poly(β-amino esters), in particular, have gained widespread use in DNA delivery owing to their ease of synthesis and biodegradability [21], but have also proved to be capable of effecting delivery of short interfering RNA (siRNA) [22–24] and mRNA [25].

**Table 1:** Comparison of clinically relevant RNA delivery platforms

Delivery vehicle	Type of RNA in clinical trials	Advantages	Disadvantages	References
Naked RNA	siRNA, ASO, mRNA	No additional materials or synthesis required	Prone to degradation Immunogenic Difficulty entering cell Poor circulation half-life	[63–65, 73–78, 101, 103, 114, 115]
Nanoparticle	siRNA, ASO, mRNA	Increased half life Protection from nucleases Aids in endocytosis and endosomal escape	Elevated risk of toxicity with introducing excipient materials	[12–37, 58–60, 82–85, 106–108, 110–113, 131, 145, 156–159]
Conjugate	siRNA, ASO	Defined chemical structure Ability to target specific receptors Limited toxicity due to lack of excipient materials	High doses required Dependent on chemical modifications for RNA stability	[38–43, 62]

*ASO* antisense oligonucleotide, *siRNA* short interfering RNA

Lipids and lipid-like materials represent the second major class of nanoparticle-based delivery vehicles for RNA. As with polymers, cationic lipids are often used to electrostatically bind the nucleic acid. Many laboratories, however, have started utilizing ionizable lipids, which are lipids that are positively charged only at acidic pH. This ionizable behavior is thought to enhance efficacy through helping with endosomal escape [26] and reducing toxicity [27] as compared with particles that remain cationic at physiological pH. Lipids are also capable of self-assembly into well-ordered nanoparticle structures, known as lipoplexes (Fig. 1b), driven by a combination of electrostatic interactions with RNA and hydrophobic interactions [28, 29]. Optimizing the formulation of lipid nanoparticles (LNPs) by addition of other hydrophobic moieties, such as cholesterol and PEG-lipid, in addition to an ionizable/cationic lipid, enhances nanoparticle stability and can significantly enhance efficacy of RNA delivery [30]. However, similarly to polymers, it was found that ionizable lipid structure is the main factor affecting efficacy of the nanoparticle. As such, one laboratory has pioneered the use of semi-automated high-throughput synthesis methods to create libraries of chemically diverse lipids and lipid-like materials for RNA delivery [31–35], resulting in highly potent nanoparticles capable of delivering a variety of RNA types to both the liver [32, 36, 37] and the lung [33] following systemic delivery in vivo.

As an alternative to nanoparticles, a more conceptually straightforward and chemically well-defined means of delivery is to directly conjugate a bioactive ligand to the RNA that will allow it to enter the cell of interest. Perhaps the most clinically advanced example of this technique is the conjugation of *N*-acetylgalactosamine (GalNAc; Fig. 1c), which targets the asialoglycoprotein receptor on hepatocytes, to siRNA [38]. Unlike many nanoparticles, which are given intravenously, GalNAc conjugates are typically dosed subcutaneously and have shown an ability to rapidly enter systemic circulation and target the liver [39]. Other conjugates, such as cholesterol [40], vitamin E [41], antibodies [42], and cell-penetrating peptides [43], have been explored in the past, although none but the specialized triantennary GalNAc–siRNA conjugate has gained any clinical traction (Table 2), suggesting the need for additional work on the design of conjugates for efficient delivery of nucleic acids.

**Table 2:** Current clinical trials involving RNA delivery

Name	Treatment	Genetic/protein target	Delivery vehicle	Administration method	Disease	ClinicalTrials.gov identifier	Phase
siRNA-EphA2-DOPC	siRNA	EphA2	Lipid nanoparticle	Intravenous infusion	Solid cancer	NCT01591356	I
TD101	siRNA	K6a	Naked (unmodified)	Intralesional injection	Pachyonychia congenita	NCT00716014	I
Atu027	siRNA	PKN3	Lipid nanoparticle	Intravenous infusion	Solid cancer	NCT00938574	I
ND-L02-s0201	siRNA	HSP47	Lipid nanoparticle	Intravenous infusion	Liver fibrosis	NCT01858935, NCT02227459	I
DCR-PH1	siRNA	Glycolate oxidase	Lipid nanoparticle	Intravenous infusion	Primary hyperoxaluria type 1	NCT02795325	I
STP705	siRNA	TGF-1β and Cox-2	Polymer nanoparticle	Intradermal injection	Hypertrophic scarring	NCT02956317	I/II
ALN-GO1	siRNA	Glycolate oxidase	Conjugate (GalNAc)	Subcutaneous injection	Primary hyperoxaluria type 1	NCT02706886	I/II
Fitusiran (ALN-AT3SC)	siRNA	Plasma antithrombin	Conjugate (GalNAc)	Subcutaneous injection	Severe hemophilia A or B	NCT02554773	I/II
ALN-CC5	siRNA	Complement component C5	Conjugate (GalNAc)	Subcutaneous injection	Paroxysmal nocturnal hemoglobinuria	NCT02352493	I/II
ALN-AS1	siRNA	ALAS-1	Conjugate (GalNAc)	Subcutaneous injection	Acute intermittent porphyria	NCT02949830	I/II
DCR-MYC	siRNA	MYC	Lipid nanoparticle	Intravenous infusion	Solid cancer	NCT02110563 NCT02314052	II/II
TKM 080301	siRNA	PLK1	Lipid nanoparticle	Liver injection Intravenous infusion Intravenous infusion	Liver cancer Liver cancer Adrenocortical carcinoma	NCT01437007 NCT02191878 NCT01262235	I I/II I/II
siG12D-LODER	siRNA	KRASG12D	Degradable polymer	Local implantation	Pancreatic cancer	NCT01676259	II
Inclisiran (ALN-PCSSC)	siRNA	PCSK9	Conjugate (GalNAc)	Subcutaneous injection	Hypercholesterolemia	NCT03060577	II
PF-655	siRNA	RTP801	Naked (modified)	Intravitreal injection	Diabetic macular edema	NCT01445899	II
SYL1001	siRNA	TRPV1	Naked (modified)	Eye drops	Dry eye syndrome	NCT02455999	II
Bamosiran (SYL040012)	siRNA	β-2 adrenergic receptor	Naked (modified)	Eye drops	Glaucoma	NCT02250612	II
QPI-1007	siRNA	Caspase 2	Naked (modified)	Intravitreal injection	Acute nonarteritic anterior ischemic optic neuropathy	NCT02341560	II/III
QPI-1002	siRNA	p53	Naked (modified)	Intravenous infusion	Prevention of acute kidney injury Delayed graft function in kidney transplant recipients	NCT02610283 NCT02610296	II III
Patisiran (ALN-TTR02)	siRNA	TTR	Lipid nanoparticle	Intravenous infusion	Familial amyloid polyneuropathy	NCT01960348	III
ISTH0036	ASO	TGF-β2	Naked (modified)	Intravitreal injection	Glaucoma	NCT02406833	I
EZN-2968 (RO7070179)	ASO	HIF1A	Naked (modified)	Intravenous infusion	Liver cancer	NCT02564614	I
LErafAON-ETU	ASO	C-raf	Lipid nanoparticle	Intravenous infusion	Advanced cancer	NCT00100672	I
AKCEA-APOCIII-LRx	ASO	ApoCIII	Conjugate (GalNAc)	Subcutaneous injection	Elevated triglycerides	NCT02900027	I
BIIB067 (IONIS-SOD1Rx)	ASO	SOD1	Naked (modified)	Intrathecal injection	Amyotrophic lateral sclerosis	NCT02623699	I
AZD5312	ASO	Androgen receptor	Naked (modified)	Intravenous infusion	Prostate cancer	NCT02144051	I
Cenersen	ASO	p53	Naked (modified)	Intravenous infusion	Myelodysplastic syndrome	NCT02243124	I
IONIS-HTT Rx	ASO	Huntingtin	Naked (modified)	Intrathecal injection	Huntington's disease	NCT02519036	I/II
IONIS ANGPTL3-LRx	ASO	ANGPTL3	Conjugate (GalNAc)	Subcutaneous injection	Elevated triglycerides/familial hypercholesterolemia	NCT02709850	I/II
AZD9150	ASO	STAT3	Naked (modified)	Intravenous infusion	Solid cancer	NCT01563302	I/II
QR-010	ASO	CFTR (causes base insertion)	Naked (modified)	Nebulization (inhaled)	Cystic fibrosis	NCT02532764	I/II
SB012	ASO	GATA-3	Naked (modified)	Enema	Ulcerative colitis	NCT02129439	I/II
AEG35156	ASO	XIAP	Naked (modified)	Intravenous infusion	Solid cancer	NCT00882869	I/II
DS-5141b	ASO	Dystrophin (exon skipping)	Naked (modified)	Subcutaneous injection	Duchenne muscular dystrophy	NCT02667483	I/II
AKCEA-APO(a)-LRx	ASO	ApoA	Conjugate (GalNAc)	Subcutaneous injection	Hyperlipoproteinemia(a)	NCT03070782	II
Apatorsen (OGX-427)	ASO	Hsp27	Naked (modified)	Intravenous infusion	Solid cancer	NCT01829113	II
IONIS-HBV Rx	ASO	HBV surface antigen	Naked (modified)	Subcutaneous injection	Hepatitis B infection	NCT02981602	II
IONIS-GCGR Rx	ASO	GCGR	Naked (modified)	Subcutaneous injection	Type 2 diabetes	NCT02824003	II
ASM8	ASO	CCR3, β-chain of IL-3, IL-5, and GM-CSF	Naked (modified)	Nebulization (inhaled)	Allergen-induced asthma	NCT00822861	II
SB010	ASO	GATA-3	Naked (modified)	Nebulization (inhaled)	Asthma	NCT01743768	II
SB011	ASO	GATA-3	Naked (modified)	Topical	Atopic dermatitis	NCT02079688	II
G4460	ASO	C-myb	Naked (modified)	Intravenous infusion	Liquid cancer	NCT00780052	II
Prexigebersen (BP1001)	ASO	Grb2	Lipid nanoparticle	Intravenous infusion	Myeloid leukemia	NCT02781883	II
IONIS-FXI Rx	ASO	Factor XI	Naked (modified)	Subcutaneous injection	Clotting disorders	NCT02553889 NCT01713361	II
Aganirsen (GS-101)	ASO	IRS-1	Naked (modified)	Eye drops	Neovascular glaucoma	NCT02947867	II/III
Eteplirsen (AVI-4658)	ASO	Dystrophin (exon skipping)	Naked (modified)	Intramuscular injection	Duchenne muscular dystrophy	NCT02255552	III
Alicaforsen	ASO	ICAM-1	Naked (modified)	Enema	Pouchitis	NCT02525523	III
Volanesorsen	ASO	ApoCIII	Naked (modified)	Subcutaneous injection	Familial chylomicronemia syndrome Familial partial lipodystrophy	NCT02658175 NCT02527343	III II/III
IONIS-TTR Rx	ASO	TTR	Naked (modified)	Subcutaneous injection	Familial amyloid polyneuropathy	NCT01737398	III
Custirsen (OGX-011)	ASO	Clusterin	Naked (modified)	Intravenous infusion	Prostate cancer Non small cell lung cancer	NCT01578655 NCT01630733	III
Lipo-MERIT	mRNA	Tumor-associated antigens	mRNA–Lipoplex	Intravenous infusion	Melanoma	NCT02410733	I
IVAC mutanome/warehouse	mRNA	Patient-specific tumor antigens	Naked mRNA	Intra-nodal	Triple negative breast cancer Melanoma	NCT02316457 NCT02035956	I
TNBC-MERIT	mRNA	Tumor-associated antigens	mRNA–Lipoplex	Intravenous infusion	Triple negative breast cancer	NCT02316457	I
CV7201	mRNA	Rabies virus glycoprotein	Naked mRNA	Intramuscular injection	Rabies	NCT02241135	I
CV8102	mRNA	RNA-based adjuvant	Naked mRNA	Intramuscular injection	RSV, HIV, rabies	NCT02238756	I
mRNA-1851	mRNA	Hemagglutinin 7 (H7) protein	ND	Intramuscular injection	Influenza A	ND	I
mRNA-1440	mRNA	Hemagglutinin 10 (H10) protein	ND	Intramuscular injection	Influenza A	ND	I
mRNA MRK-1777	mRNA	Vaccine	ND	Intramuscular injection	Undisclosed	ND	I
mRNA AZD-8601	mRNA	VEGF-A	Naked (modified)	Intradermal	Cardiovascular disease	NCT02935712	I
mRNA-1325	mRNA	Viral antigenic proteins	Lipid nanoparticle	Intramuscular injection	Zika	NCT03014089	I/II
CV9103	mRNA	Tumor-associated antigens	Naked mRNA	ND	Prostate cancer	NCT00831467	I/II
	mRNA	Tumor-specific antigens	Naked mRNA	Autologous dendritic cell therapy	Prostate cancer patients	NCT01197625	I/II
AGS-004	mRNA	Vaccine	Naked mRNA	Autologous dendritic cell therapy	HIV infections	NCT01069809, NCT02707900	I II
	mRNA	CT7, MAGE-A3, and WT1	Naked mRNA	Autologous dendritic cell therapy	Multiple myeloma Acute myeloid leukemia	NCT01995708 NCT01686334	I II
AGS-003-LNG	mRNA	Tumor-specific antigens	Naked mRNA	Autologous dendritic cell therapy	Non-small cell lung cancer	NCT02662634	II
iHIVARNA-01	mRNA	HIV target antigens	Naked mRNA	Intranodal route	HIV infections	NCT02888756	II
AGS-003	mRNA	Tumor-specific antigens	Naked mRNA	Autologous dendritic cell therapy	Renal cell carcinoma	NCT01482949 NCT00678119 NCT01582672	II III

*ASO* antisense oligonucleotide, *mRNA* messenger RNA, *siRNA* short interfering RNA, *ND* not disclosed

### RNA modifications

Equally important for effective nucleic acid delivery are chemical modifications made to the RNA itself, which can impart degradation resistance to the RNA [44] and render them unrecognizable by the immune system [45]. This is true of both conjugate delivery systems, which leave the RNA exposed immediately upon injection, as well as nanoparticulate delivery systems, which must at some point expose the RNA to intracellular immune receptors. RNAs can be modified by means of chemical alterations to the ribose sugar (of particular importance is the 2′ position [45, 46]), the phosphate linkage and the individual bases (Fig. 1d) [47–50]. RNAs delivered through nanoparticles, discussed later, are also typically modified in order to avoid recognition by endosomally expressed pattern recognition receptors [51]. With few exceptions, modified RNAs are the gold standard in clinical trials (Table 2). The degree to which the RNA can be modified and still retain its potency depends, to a large extent, on the nature of the nucleic acid and its mechanism of action. For instance, short RNAs such as siRNAs, which rely on the relatively robust RNA-induced silencing complex (RISC) [52], can typically be heavily modified. By contrast, large mRNAs, which must be effectively translated by ribosomes, are more sensitive to modifications and utilize naturally occurring RNA modification such as pseudouridine and 5-methylcytidine substitution [53]. Indeed, recent studies have shown that base modification of mRNA can actually *decrease* potency in certain situations [54], whereas chemical modification in siRNAs is almost ubiquitously applied for in vivo use [55].

## Applications of RNA-based gene/protein modulation

### Protein downregulation—siRNA, ASOs, and microRNA

**Fig. 2:**
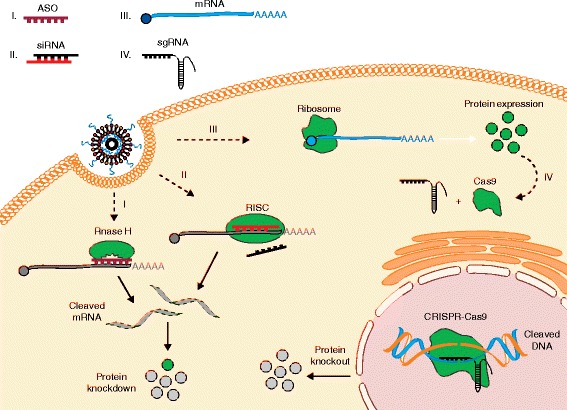
Regulation of gene and protein expression using RNA. Once delivered into the cells, RNA macromolecules can utilize diverse intracellular mechanisms to control gene and protein expression. (I) Hybridization of antisense oligonucleotides (ASOs) to a target mRNA can result in specific inhibition of gene expression by induction of RNase H endonuclease activity, which cleaves the mRNA–ASO heteroduplex. (II) Short interfering RNA (siRNA) is recognized by the RNA-induced silencing complex (RISC), which, guided by an antisense strand of the siRNA, specifically binds and cleaves target mRNA. (III) In vitro transcribed mRNA utilizes the protein synthesis machinery of host cells to translate the encoded genetic information into a protein. Ribosome subunits are recruited to mRNA together with a cap and poly(A)-binding proteins, forming a translation initiation complex. (IV) In the CRISPR–Cas9 system, co-delivery of a single guide RNA (sgRNA) together with the mRNA encoding the Cas9 DNA endonuclease allows site-specific cleavage of double-stranded DNA, leading to the knockout of a target gene and its product. CRISPR, clustered regularly interspaced short palindromic repeats

In simplistic terms, disease-relevant proteins can be altered in one of two ways: upregulated or downregulated. The use of RNAs to selectively downregulate proteins experienced a paradigm shift following the discovery of siRNA by Fire and colleagues [56]. Short interfering RNAs are typically 21–23 base-pairs in length and can selectively bind and degrade complementary mRNA through the RISC (Fig. 2) [57]. After almost two decades of research, siRNA-based therapies represent one of the more clinically advanced platforms for RNA drugs. Alnylam Pharmaceuticals, in particular, has several siRNA drugs in clinical trials. Their most advanced drug, also one of the most advanced siRNA therapeutics, patisiran, is a LNP containing siRNA against mutant transthyretin for the treatment of transthyretin amyloidosis [58]. Patisiran is currently in phase III of clinical trials [59], having shown significant dose-dependent knockdown, with minimal adverse events, in phase II trials [60], and other companies have also invested in the use of lipoplex-based siRNA drugs (Table 2). Increasingly, however, Alnylam and others have reported significant progress with the GalNAc conjugate technology (Table 2). Despite Alnylam’s recent decision to discontinue development of revusiran, a GalNAc–siRNA conjugate drug that also treats transthyretin amyloidosis [61], the company has several more GalNAc conjugates in its pipeline that utilize a newer ‘enhanced stabilization chemistry’ [62] that could address the issues that led to the removal of revusiran from clinical trials [61]. Surprisingly, some of the current clinical trials utilize naked, albeit chemically modified, siRNAs. Almost all of these naked siRNAs are delivered locally (Table 2), reducing the risk of RNA degradation and systemic immune activation compared with that associated with systemic delivery. An intriguing use of naked siRNA is Silenseed’s siG12D LODER, which encapsulates siRNA targeted against the KRAS oncoprotein in an implantable and degradable polymeric matrix for the treatment of pancreatic cancer [63, 64]. However, there is concern that the positive effects of such treatments might in some cases be mediated by the induction of non-specific and immunological mechanisms such as siRNA binding to toll-like receptors [65].

Despite its significant presence in clinical trials, siRNA is not the only, or even the first, RNA drug to be investigated for protein knockdown at the clinical stage. The first RNA drugs widely used in clinical trials were anti-sense oligonucleotides (ASOs). Like siRNA, ASOs are designed to block protein translation through Watson–Crick base-pairing with the target mRNA [66] and can be modified to improve stability [67]. The ASOs, however, inhibit protein production through a variety of mechanisms, such as sterically blocking ribosome attachment or eliciting RNase-H activation [68]. They can also promote exon skipping (a form of RNA splicing which leaves out faulty exons), which allows for the deletion of faulty sequences within proteins [69], and, in some cases, can even lead to protein upregulation, which could be used therapeutically in diseases where certain genes are repressed [70]. An additional utility of ASOs is their ability to enter cells without the use of a transfection reagent, although this uptake does not always lead to therapeutic action [71]. Four ASOs have been clinically approved, all of which are chemically modified and used without a delivery vehicle, representing the only RNA drugs for protein modulation to be cleared by the FDA so far. The most recent, Spinraza (nusinersen), is injected intrathecally to treat spinal muscular atrophy [72]. It joined Exondys 51 (eteplirsen), an intravenously infused ASO for treatment of Duchenne muscular dystrophy [73], Vitravene (fomivirsen), an intravitreally injected ASO indicated for the treatment of ocular cytomegalovirus [74], and Kynamro (mipomersen), which is injected subcutaneously and targets mRNA encoding apolipoprotein B for the treatment of hypercholesterolemia [75, 76]. There are still several ASOs in clinical trials, the majority of which are delivered without a vehicle (Table 2). Of particular interest are studies by Ionis Pharmaceuticals utilizing a GalNAc–ASO conjugate similar to that developed by Alnylam to deliver siRNA. Optimism from such approvals and clinical studies has also led researchers to continue investigation of ASOs to treat diseases such as amyotrophic lateral sclerosis (ALS) [77] and spinocerebellar ataxia [78].

An emerging, albeit less clinically advanced, RNA-based platform for protein knockdown is microRNA (miRNA). Endogenous microRNAs are non-coding RNAs that act as key regulators for a variety of cellular pathways, and are often downregulated in diseases [79]. Thus, exogenous microRNAs, or microRNA mimics, delivered therapeutically could be used to knockdown several proteins simultaneously, which is particularly useful in diseases such as cancer where having a single disease-relevant target is rare [80]. It is also worth noting that a rare subset of microRNAs is thought to enhance protein production, and that targeting of gene-suppressing microRNAs using ASOs could also be used to increase protein production [81]. The majority of current clinical trials involving microRNA are screens to investigate microRNA involvement in certain diseases, although there are several ongoing animal studies utilizing microRNA delivery. Examples include the use of LNPs to treat a mouse model of colorectal cancer [82], and polymeric nanoparticles to deliver microRNA to the heart to treat fibrosis [83]. The first microRNA mimic therapy to enter clinical trials was MRX-34—a liposomal-encapsulated microRNA mimic from Mirna Therapeutics meant to treat a variety of cancers [84]. However, the company terminated the study earlier in 2017 after reports of several immune-related severe adverse events [85]. The fact that the adverse events were immunological in character further highlights the importance of RNA modification for clinical applications, as such modifications remain one of the most important means of evading immune detection for RNA drugs. Chemical modification of miRNA mimics in particular, however, might prove challenging owing to the complex nature of miRNA-induced gene regulation [86].

### Protein overexpression—mRNA

Expression of disease-relevant proteins can be achieved by intracellular delivery of plasmid DNA (pDNA) or messenger RNA (mRNA). Application of DNA or mRNA as protein intermediate enables expression of virtually any desired protein inside the host cells and tissues. This approach can address formulation and delivery challenges encountered with protein-based drugs, especially those aimed at intracellular targets [87]. mRNA-based therapeutics in particular offer several advantages over pDNA, including rapid and transient protein production, no risk of insertional mutagenesis, and greater efficacy of non-viral delivery by virtue of mRNA cytoplasmic activity (Fig. 2) [88]. Since the first pre-clinical studies in the 1990s, mRNA technology has greatly developed and now holds the potential to revolutionize vaccination, protein-replacement therapies, and treatment of genetic diseases, consequently gaining a considerable level of interest among the scientific community and biotech industry [53].

The delivery of mRNA therapeutics has been facilitated by significant progress in maximizing the translation and stability of mRNA, preventing its immune-stimulatory activity and the development of in vivo delivery technologies, some of which are discussed below. The 5′ cap and 3′ poly(A) tail are the main contributors to efficient translation and prolonged half-life of mature eukaryotic mRNAs. Incorporation of cap analogs such as ARCA (anti-reverse cap analogs) and poly(A) tail of 120–150 bp into in vitro transcribed (IVT) mRNAs has markedly improved expression of the encoded proteins and mRNA stability [89, 90]. New types of cap analogs, such as 1,2-dithiodiphosphate-modified caps, with resistance against RNA decapping complex, can further improve the efficiency of RNA translation [91]. Replacing rare codons within mRNA protein-coding sequences with synonymous frequently occurring codons, so-called codon optimization, also facilitates better efficacy of protein synthesis and limits mRNA destabilization by rare codons, thus preventing accelerated degradation of the transcript [92, 93]. Similarly, engineering 3′ and 5′ untranslated regions (UTRs), which contain sequences responsible for recruiting RNA-binding proteins (RBPs) and miRNAs, can enhance the level of protein product [53, 94]. Interestingly, UTRs can be deliberately modified to encode regulatory elements (e.g., K-turn motifs and miRNA binding sites), providing a means to control RNA expression in a cell-specific manner [95]. Some of the previously discussed RNA base modifications such as N1-methyl-pseudouridine have not only been instrumental in masking mRNA immune-stimulatory activity but have also been shown to increase mRNA translation by enhancing translation initiation [96, 97]. In addition to their observed effects on protein translation, base modifications and codon optimization affect the secondary structure of mRNA, which in turn influences its translation [98]. Understanding the importance of, and the ability to predict, the folding structure of mRNA could aid engineering of mRNA therapeutics—however, the accuracy of available prediction tools is currently limited. Despite the plethora of carriers studied for other types of RNA drugs, mRNA molecules are significantly larger (600–10,000 kDa) than the previously discussed siRNAs (~14 kDa) and ASOs (4–10 kDa), which poses an additional challenge for delivery of mRNA therapeutics [99]. Accommodation of large and charged mRNAs into nanoparticles and their effective intracellular release has been shown to require fine-tuning of existing formulations and the development of a new-generation of biomaterials with higher potency [36, 37].

Therapeutic applications of mRNA that are currently being explored are vaccinations against cancer and infectious disease, protein-replacement therapy, and gene editing. A comprehensive list of ongoing clinical trials involving mRNA can be found in Table 2. mRNA vaccines are in the most-advanced stages of clinical development, following in the footsteps of competing DNA and protein-based technologies. Synthetic mRNA vaccines allow simultaneous delivery of a wide variety of antigens and are both faster and easier to manufacture at low cost in comparison with other systems, enabling a more-rapid response towards emerging pathogens [100]. Additionally, immune responses generated by naked mRNA can be beneficial for vaccination purposes [101, 102]. Immunization against infectious diseases using ex vivo mRNA-transfected dendritic cells (DCs) is now being pursued in clinical trials and has demonstrated good safety profiles and ability to induce antigen-specific T-cell responses [103].

Another RNA vaccination approach is the use of self-amplifying mRNA replicons that have been developed to extend the duration and magnitude of antigen expression as well as boost the immune response [104, 105]. In a recent study, replicon vaccines formulated into nanoparticles comprising repeatedly branched dendrimer (tree-like) molecules have generated protective immunity against a broad spectrum of lethal pathogens, including Zika, Ebola and influenza viruses [106]. Conventional, modified mRNAs are also being explored for vaccination [105]. Lipid-nanoparticle-encapsulated mRNAs encoding pre-membrane and envelope glycoproteins of Zika virus have recently been reported to elicit potent and durable neutralizing antibody responses in mice and non-human primates against the virus after intradermal administration [107]. Moreover, expression of modified mRNA encoding broadly neutralizing antibody in the liver, after systemic administration of mRNA–LNPs, has protected humanized mice against HIV-1 challenge [108]. Cancer mRNA vaccines have experienced accelerated development and clinical translation driven by the success of cancer immunotherapy. The majority of approaches tested in clinical trials employ adoptive-transfer of DCs transfected with mRNAs coding for tumor-specific antigens (TSAs) and immunomodulation of T cells with mRNAs expressing chimeric antigen receptors (CARs) or TSAs [109]. In addition, direct intradermal and systemic administration of LNP-formulated mRNAs coding for tumor-specific antigens is currently being investigated in the clinic for induction of T-cell immune responses [100, 110, 111].

By contrast, most mRNA-based protein replacement therapies are still in the preclinical stages of development and involve supplementation of deficient or aberrant proteins as well as modulation of cell behavior by expression of exogenous proteins. The in vivo efficacy of RNA–protein therapy has been demonstrated for a number of diseases. The majority of the studies preferentially target the liver owing to the well-established and efficient methods for RNA delivery into liver tissue. Therapeutically relevant amounts of human FIX (hFIX) protein were reached and sustained physiological activity for 4–9 days upon a single intravenous dose of hFIX mRNA-loaded LNPs in mice with hemophilia B [112, 113]. Similarly, LNPs formulated with mRNA encoding erythropoietin (Epo) have been shown to elicit a systemic physiological response in large animals, including pigs and nonhuman primates [93]. Therapeutic effects of mRNA have also been demonstrated in other tissues. Lung delivery of surfactant protein B (SP-B) mRNA protected mice from respiratory failure [114], whereas myocardial injection of RNAiMAX-formulated mRNA, encoding human vascular endothelial growth factor A (VEGF-A), improved heart regeneration after myocardial infarction in mice [115]. Based on this notion, Astra Zeneca partnered by Moderna has launched a phase I clinical trial for local delivery of VEGF mRNA, starting January 2017 [116]. Pre-clinical studies have demonstrated the translational potential of mRNA-based protein therapy for both secreted and intracellular protein targets. However, treatment of chronic diseases might carry an elevated risk of toxicity, associated with the repeated mRNA–LNP administrations required to sustain therapeutic levels of protein. Using mRNA for delivery of gene editing tools could address this challenge and is discussed below.

## Gene editing

The RNA-based technologies discussed above constitute a powerful means to transiently repress or overexpress the expression of genes. By contrast, therapeutic gene editing entails replacement or alteration of gene expression by introducing site-specific modifications into the genome of cells, including correction of deleterious or introduction of protective mutations [117]. While the majority of current gene editing efforts are focused on treatment of monogenic disorders, caused by deleterious changes in a single gene, the expansion of gene editing and delivery tools makes the treatment of complex polygenic diseases such as cardiovascular diseases [118] and antiviral therapies [119], as well as editing the epigenome, more feasible [120]. The discovery of RNA-guided DNA endonucleases such as Cas9 associated with CRISPR (clustered regularly interspaced short palindromic repeats), elements composing the prokaryotic adaptive immune system [121], equipped scientists with an easy-to-use and efficient platform to alter genomic information [122]. So-called CRISPR–Cas systems rely on Watson–Crick base-pairing between a single guide RNA (sgRNA) and a corresponding DNA target site followed by a distinct protospacer-adjacent motif (PAM), a 3–5-nucleotide DNA sequence required for binding of Cas9 and cleavage of the target sequence, in order to introduce a double-stranded break (DSB) into a DNA molecule [123]. DSBs can be repaired by the cells using non-homologous end joining (NHEJ) and homology-directed repair (HDR). NHEJ results in stochastic insertions and deletions (‘indels’) causing permanent gene knockout, whereas HDR occurs in the presence of a DNA template containing homology to regions flanking the DSB site, leading to incorporation of desired changes encoded in the repair template into the genome [124]. A combination of DSBs can also be used to edit multiple loci by employing different sgRNAs [125, 126].

To date, the most widely used and well characterized gene-editing technology is the CRISPR–Cas9 system with an effector domain originating from *Streptococcus pyogenes* (SpCas9). Direct in vivo delivery of spCas9 to diseased cells has recently been used to correct mutations in genes in animal models of Duchenne muscular dystrophy (*mdx*) [127–129], hereditary tyrosynemia type I (*fah*) [130, 131], and lethal metabolic liver disease (*oct*) [132] and to reduce blood cholesterol in chimeric mice with humanized liver by knockout of *PCSK9* [133]. Ex vivo editing with spCas9 has been applied to human hematopoietic stem cells in order to correct sickle cell anemia caused by mutation in the gene encoding β-globin, as well as to deplete T cells of expression of *CCR5* to trigger anti-HIV protection or to deplete PD-1 to boost anti-cancer therapy [134]. Despite positive outcomes, these studies have revealed limitations of the CRISPR–Cas9 system relevant for clinical translation, including (1) imperfect DNA-targeting specificity leading to off-target effects [135], (2) low efficiency of genome editing using HDR [136], and (3) challenging delivery of CRISPR–Cas9 components using both viral and non-viral methods [137].

The DNA-targeting specificity of CRISPR–Cas9 can be improved by combining optimized design and synthesis of guide RNAs. In particular, sgRNAs shorter than 20 nucleotides and containing 5′ mismatches have shown fewer off-target effects [138, 139], whereas chemically synthesized sgRNAs bearing base modifications at the 5′ and 3′ ends have demonstrated increased on-target efficacy [140]. Furthermore, improved types of spCas9, such as high-fidelity spCas9-HF1 [141] or enhanced-specificity eSpCas9 [142], have been engineered by introducing specific mutations into spCas9 based on interactions between a spCas9–gRNA complex and DNA. New RNA-guided nucleases, such as Cpf1 from *Acidaminococcus* sp. (AsCpf1), with the capability to edit the genome of mammalian cells have been discovered recently [143, 144]. Cpf1 nuclease mRNA (~1.3 kb) is significantly smaller than Cas9, with a different PAM requirement and inherently higher DNA specificity than spCas9, which makes it attractive for clinical use. Off-target effects can be also limited by decreasing the cellular presence of spCas9 through conditions favoring transient over long-lasting expression, which can be accomplished by optimizing the delivery method [140, 145].

Obtaining a better efficiency of genome editing by HDR will be necessary to address genetic diseases demanding a high level of therapeutic product, especially when edited cells do not display a positive change in fitness and outcompete their diseased counterparts over time [117]. The efficiency of correction by HDR can be significantly improved by designing an asymmetric single-stranded DNA template that anneals to the non-target DNA strand, which is the first to be released from the Cas9–DNA complex [146]. In addition, a number of studies have reported better HDR efficacy by using CRISPR–Cas9 in combination with small-molecule inhibitors of NHEJ, such as DNA ligase IV or DNA-dependent protein kinase inhibitors [147, 148]. Alternatively, HDR can be enhanced by agonists of proteins crucially involved in homologous recombination such as Rad51 [149]. Recently, other methods of gene editing with CRISPR–Cas9, called homology-independent targeted integration (HITI), have been developed, which exploit the NHEJ repair mechanism for gene knock-ins [150]. HITI donor templates are designed to ensure robust gene integration only when inserted in the correct direction as otherwise the target DNA would undergo additional cleavage by Cas9. This method has demonstrated higher in vitro efficacy of transgene insertion in comparison with HDR-dependent editing, but so far when conducted in vivo it reached only 3–10% of the knock-in efficiencies.

Intracellular delivery of CRISPR-based agents poses one of the most significant challenges for therapeutic genome editing owing to the number of essential components. CRISPR–Cas9 components can be delivered as DNA, RNA, RNA–protein complex (RNP), or a combination of these macromolecules. These macromolecules are not able to spontaneously enter the cells, relying on the use of delivery vehicles such as viral vectors, nanoparticles, or physical and mechanical delivery methods like nucleofection, cell squeezing, or lipofection that utilize electric field, mechanical force, or cationic lipids for temporary disruption of the cell membrane [151]. The latter are primarily suited for therapeutic ex vivo gene editing, while viral vectors and nanoparticles are mainly used for in vivo gene therapy [152].

Viral delivery of CRISPR–Cas9 has been explored using lentivirus, adeno-virus, and adeno-associated virus (AAV) [137]. AAVs are most widely used for gene therapy clinical trials due to their ability to transduce different cell types and tissues and their low risk of genomic integration and low immunogenicity [153]. However, AAV-limited packaging capacity (~4.5 kb) makes it impossible to accommodate all the components of CRISPR–spCas9, including sgRNA and a donor DNA template, into a single AAV. Noteworthy is that a host immune response to AAV-CRISPR–Cas9 has been observed in mice, elicited by Cas9 immunogenicity and possibly aggravated by its prolonged expression [154].

Complementary to the viral systems, an abundance of nanoparticles comprising various bio-compatible materials are being developed for delivery of CRISPR–Cas9. As with their use in protein modulation, nanoparticles for gene editing have demonstrated high loading capacity for nucleic acid cargos, ability to modify payload bio-distribution and pharmacokinetics through active targeting and formulation, as well as simplicity of manufacturing with a high level of control over their physicochemical parameters, such as size/shape and kinetics of payload release [155]. Nanoparticle-based mRNA delivery of CRISPR–Cas components is therapeutically attractive owing to the transient nature of mRNA expression, no risk of genomic integration and mRNA cytoplasmic activity, alleviating the need to overcome the nuclear barrier in comparison with pDNA (Fig. 2). To date, nanoparticle-mediated delivery of spCas9 mRNA has been used in combination with AAVs encoding a sgRNA and a repair template to induce repair of the *Fah* gene in a hereditary tyrosinemia in adult animals [131]. The efficiency of correction was >6% of hepatocytes after a single application, much higher than with a hydrodynamic injection of pDNA (0.4%) previously reported for the same disease [130]. Similarly, lung delivery of mRNA encoding zinc-finger nucleases complexed into chitosan-coated nanoparticles, used in combination with an AAV6-expressing donor template, resulted in correction of the gene encoding surfactant protein B in mice with SP-B deficiency and extended their survival [156]. Interestingly, the combination of mRNA nanoparticle with the virus was superior to AAV alone, reaching HDR rates in lung cells of ~9%. Recently, a study described the synthesis and development of zwitterionic amino lipids, composed of a sulfobetaine head group and a polyamine linker with hydrophobic tails, that were used to formulate nanoparticles capable of simultaneous in vivo delivery of Cas9 mRNA and sgLoxP to induce expression of floxed tdTomato in the liver, kidneys, and lungs of LSL-TdTomato mice [157]. This study shows the potential of the nanoparticle–RNA platform to accommodate multiple components of CRISPR–Cas9 into a single carrier, and could possibly be extended to also include a donor template. Lipid and polypeptide nanoparticles have also been used to deliver RNA–protein complex of Cas9 and sgRNAs, which is another promising strategy to ensure the transient cellular presence of Cas9, significantly reducing off-target effects [158, 159]. However, the therapeutic potential of in vivo RNP delivery has yet to be demonstrated.

## Conclusions

After over two decades of development, RNA therapeutics has become a clinical reality. The design and chemistries used to synthesize siRNAs, ASOs, and mRNAs have advanced to a point where they enable adequate stability and immune evasion, while at the same time allowing the maintenance of efficacy and specificity. The delivery technologies have also greatly progressed thanks to the discovery of potent and bio-compatible materials, aided by high-throughput screening technologies. Despite recent setbacks surrounding withdrawal of Alnylams’s siRNA–GalNac conjugate [160] and Curevac’s first mRNA vaccine [100] from clinical trials, nucleic acid-based therapeutics continue to progress, as highlighted by the approval of four ASOs by the FDA [159] and more RNA candidate drugs with improved chemical modifications entering advanced stages of human trials (Table 2). In addition, the enormous excitement surrounding CRISPR–Cas genome editing and its transformational impact on biomedical sciences has spurred the development of RNA-based delivery approaches to facilitate clinical translation of CRISPR–Cas technology. The first US-based human trial conducted by the University of Pennsylvania will use CRISPR–Cas9 ex vivo to knock out the genes encoding PD1 and T-cell receptor alpha/beta in T cells isolated from cancer patients for cancer therapy [161]. The leading CRISPR biotech companies such as CRISPR Therapeutics [162], Editas Medicine [163], and Intellia Therapeutics [164] have programs in advanced pre-clinical stages of development in their portfolios and will likely soon follow the clinical route. These companies mostly focus on disorders affecting liver, lung, and hematopoiesis, while developing both ex vivo and in vivo delivery approaches utilizing AAVs, LNPs, and RNPs [162–164]. This highlights that safety and delivery remain the major challenges for RNA-based drugs, especially for RNA–protein and CRISPR–Cas therapies, and will be shaping the scope of upcoming clinical trials. Undoubtedly, the field of RNA therapeutics is currently undergoing a major expansion, and the potential for using RNA drugs for personalized medicine and immunotherapy as well as to address genetic, infectious, and chronic diseases will ensure the continued development of RNA therapeutics for years to come.

## Abbreviations

AAV: Adeno-associated virus
ARCA: Anti-reverse-cap analog
AsCpf1: Cpf1 nuclease derived from *Acidaminococcus* species
ASO: Antisense oligonucleotide
CAR: Chimeric antigen receptor
CRISPR: Clustered regularly interspaced short palindromic repeats
DC: Dendritic cell
DSB: Double-strand break
EPO: Erythropoietin
HDR: Homology-directed repair
hFIX: Human factor IX
HITI: Homology-independent targeted integration
LNP: Lipid nanoparticle
miRNA: MicroRNA
NHEJ: Nonhomologous end joining
PAM: Protospacer-adjacent motif
RBP: RNA-binding protein
RISC: RNA-induced silencing complex
RNP: RNA–protein complex
sgRNA: Short guide RNA
siRNA: Short interfering RNA
SP-B: Surfactant protein B
spCas9: Cas9 nuclease derived from *Streptococcus pyogenes*
TSA: Tumor-specific antigen
UTR: Untranslated region
VEGF-A: Vascular endothelial growth factor A
